# Video-rate dual-modal photoacoustic and fluorescence imaging through a multimode fibre towards forward-viewing endomicroscopy

**DOI:** 10.1016/j.pacs.2021.100323

**Published:** 2021-12-31

**Authors:** Tianrui Zhao, Michelle T. Ma, Sebastien Ourselin, Tom Vercauteren, Wenfeng Xia

**Affiliations:** School of Biomedical Engineering and Imaging Sciences, King’s College London, 4th Floor, Lambeth Wing St Thomas Hospital, London SE1 7EH, United Kingdom

**Keywords:** Photoacoustic imaging, Fluorescence imaging, Endoscopy, Multimode fibre, Multi-modal imaging

## Abstract

Multimode fibres (MMFs) are becoming increasingly attractive in optical endoscopy as they promise to enable unparallelled miniaturisation, spatial resolution and cost. However, high-speed imaging with wavefront shaping has been challenging. Here, we report the development of a video-rate dual-modal photoacoustic (PA) and fluorescence microscopy probe with a high-speed digital micromirror device (DMD) towards forward-viewing endomicroscopy. Optimal DMD patterns were obtained using a real-valued intensity transmission matrix algorithm to raster-scan a 1.5 μm-diameter focused beam at the distal fibre tip for imaging. The PA imaging speed and spatial resolution were varied from ∼2 to 57 frames per second and from 1.7 to 3 μm, respectively. Further, high-fidelity PA images of carbon fibres and mouse red blood cells were acquired at unprecedented speed. The capability of dual-modal imaging was demonstrated with phantoms. We anticipate that with further miniaturisation of the ultrasound detector, this probe could be integrated into medical needles to guide minimally invasive procedures.

## 1 Introduction

Optical endoscopy is widely used for visualising internal tissues such as the gastrointestinal tract, the airways and the uterus [1]. However, conventional optical endoscopy only provides morphological information of superficial tissue, and thus biopsies are still required to achieve definitive histopathological diagnosis. Owing to the rapid development of optical imaging techniques, several optical endoscopy modalities have emerged as promising optical alternatives to biopsy, including endoscopic optical coherence tomography (EOCT) [2], [3], [4], fluorescence endoscopy (FM) [5], [6], confocal endomicroscopy (CEM) [7], [8] and PA endoscopy (PAE) [9], [10], [11], [12]. EOCT provides real-time 3D images of tissues with microscopic scale (cellular) structural information derived from optical scattering of biological tissue, however, measuring functional information such as blood oxygen saturation with EOCT is challenging. FM and CEM provide anatomical and pathological information of tissues and cells, but it is difficult to perform in-depth imaging. PAE provides 3D cellular-level imaging of internal organs in real-time, with contrast that are derived from optical absorption by intrinsic tissue chromophores such as haemoglobin, lipids, and extrinsic contrast agents [4], [11], [13], [14], [15]. Utilising excitation light with multiple wavelengths, multispectral PAE images can be acquired to obtain spatial distributions of absorbing chromophores and contrast agents [16], [17]. It is therefore ideally suited to visualise changes in vascular morphology, and blood oxygenation and metabolism that are known to be associated with tumour development [16], [17], [18].

Most of the current PAE probes are based on side-viewing configurations, with which volumetric images of tissue are acquired by pull-back of the rotating probes. These probes are useful for visualising hollow structures such as intravascular [9], [19], [20], [21] and gastrointestinal tract [10] where circumferential scanning is required. In the past few years, forward-viewing PAE probes have attracted significant research interests, as they could be more convenient for use in various clinical applications such as optical biopsy and tumour margin assessment compared to side-viewing probes [22], [23], [24]. Moreover, a forward-viewing probe could also provide visualisation of critical tissue structures during minimally invasive procedures in clinical contexts including peripheral nerve blocks and foetal interventions [25], [26], [27]. With a PA microscopy implementation, a MEMS or a galvanometer mirror system was used to scan a focused laser beam at the proximal tip of a fibre bundle into individual cores, whilst the generated ultrasound (US) A-scans were received by a single-element ultrasound (US) transducer as reported in a study by Hajireza et al. [22]. The imaging capability was demonstrated with mouse ears *in vivo*, however, the lateral resolution of the optical-resolution PAE probe was 7–8μm, limited by the large separation between individual cores of the fibre bundle [22]. Forward-reviewing PAE probe in PA tomography mode via a coherent fibre bundle was extensively studied by the group of Beard from UCL with dichromatic Fabry–Perot sensors for US detection and the system development has undergone several iterations [24], [28]. In 2018, Ansari et al. developed a PAE system by coating a transparent Fabry–Perot sensor at the tip of a 3.2 mm-diameter rigid fibre bundle and raster-scanning an interrogation laser beam through the bundle for US detection [24]. In 2020, the same group developed a flexible PAE probe with a diameter of 7.4 mm by interrogating a Fabry–Perot sensor through a flexible fibre bundle with a miniature optical relay system [28]. With these probes, high-quality 3D vasculature images of duck embryo and human placenta *ex vivo* were obtained. However, the diameters of these probes were still too large to be integrated into a biopsy needle and the imaging speed was insufficient for clinical operation (100 s to 25 min per image).

Recently, MMFs have shown great promise for use in forward-reviewing optical endoscopy owing to the rapid advances of optical wavefront shaping technology [29], [30], [31], [32], [33], [34], [35], [36], [37], [38]. Compared to coherent fibre bundles, MMFs are much more cost effective. Further, as the sizes of optical foci through a MMF are limited by optical diffraction, higher effective pixel density and achievable spatial resolution can be achieved with a MMF [39]. As such, high-resolution MMF-based probes can be readily miniaturised for integration into medical needles. With MMF-based optical endoscopy, a focused light beam is raster-scanned at the distal tip of a MMF via wavefront shaping with the knowledge of the light transmission characteristics of the MMF, which are usually achieved with transmission matrix-based methods [32], [40] or digital optical phase conjugate [35], [37] approaches using a liquid-crystal spatial light modulator (LC-SLM). In the past decade, several groups have developed ultrathin endoscopes based on MMFs and wavefront shaping for a wide range of biomedical imaging modalities including wide-field microscopy [30], confocal [31], fluorescence [29], [33], two-photon [36], Raman [38], PA imaging [37], [41] and multi-modal imaging probes [42]. However, due to the slow rates of LC-SLM (∼100Hz) and data acquisition, the fastest MMF-based PAE system reported in literature required 30 s for the acquisition of a single PA image frame comprising 1800 pixels [42], which hinders its clinical translation. In contrast to LC-SLMs, DMDs have a much higher frame rate of 22.7 kHz and consist of large arrays of micromirrors, offering binary amplitude modulations by switching ‘ON’ or ‘OFF’ micromirrors. High-speed fluorescence imaging of a mouse brain *in vivo* through a MMF using DMDs was demonstrated with a frame rate of 3.5 fps for 7000-pixel images [29], which could be further improved to 7–15 fps with sub-sampling [34].

In this work, we developed a high-speed, MMF-based PA and fluorescence microscopy system using a DMD. The performance of two types of MMFs, a step-index (STIN) and a gradient-index (GRIN) fibre with similar core diameters, were compared and the GRIN fibre was preferred due to its more uniform intensity distribution of the generated optical foci. As the scanning step size can be easily varied using a DMD, the lateral resolution and the imaging speed of the developed imaging system were scalable. With a carbon fibre phantom, the measured spatial resolution of the PA images ranged from 1.7 to 3μm with the scanning step size varying from 0.5 to 2.5μm, and the corresponding imaging speed with a field-of-view of 50 μm × 50 μm varied from ∼2 to 57 fps. The ability of the developed imaging probe was further showcased by imaging mouse red blood cells (RBCs) at a frame rate up to ∼20 fps. Finally, the capability of dual-modal forward-reviewing PA and fluorescence imaging was demonstrated with phantoms comprising carbon fibres and fluorescence microspheres.

## 2 Materials and methods

### 2.1 Imaging system

**Fig. 1:**
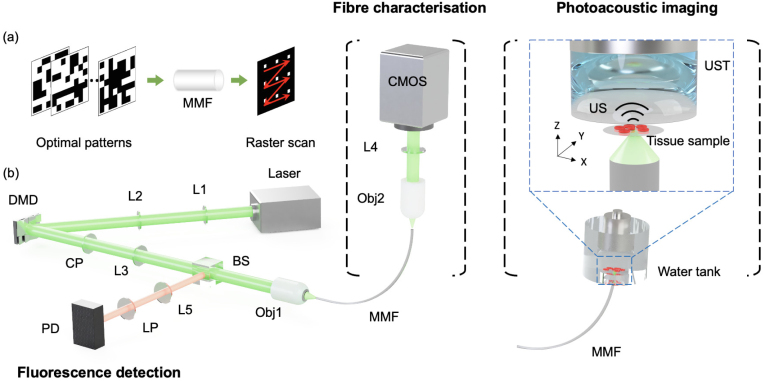
Schematic diagrams of the principle and configuration of the video-rate dual-mode forward-viewing photoacoustic and fluorescence endo-microscopy system. (a) The principle of raster-scanning of a focused light beam through a multimode fibre (MMF) for endo-microscopy imaging by sequentially displaying optimal DMD patterns via optical wavefront shaping. (b) Configuration of the imaging system. After fibre characterisation, the characterisation module is replaced by the photoacoustic imaging module, and the imaging target is placed at the optical focal plane. Photoacoustic signals are detected by the ultrasound transducer (UST) and excited fluorescence signals are captured by a photodetector (PD) through the MMF. L2, tube lens (f = 50 mm); L3-5, tube lenses (f = 100 mm); DMD: digital micromirror device; CP, circular polariser; LP, longpass Filter (cut-on wavelength: 550 nm); CMOS: complementary metal–oxide–semiconductor camera; US, ultrasound; Obj1-2: Objective lenses.

A schematic diagram of the imaging system is shown in Fig. 1. A pulsed laser (532 nm, 2 ns, 50 kHz, SPOT-10-200-532, Elforlight, Daventry, United Kingdom) was used as the light source for both PA and fluorescence imaging. A DMD with 768 × 1080 pixels (DLP7000, Texas Instruments, Texas, USA) was used to project binary patterns onto the proximal end of a MMF via a tube lens (AC254-050-A-ML, Thorlabs, New Jersey, USA), a circular polariser (CP1L532, Thorlabs, New Jersey, USA) and an objective (20×, 0.4 NA, RMS20X, Thorlabs, New Jersey, USA). Two types of MMFs including a STIN 105 μm, 0.22 NA) and a GRIN (100 μm, 0.29 NA) fibre with the same length of 20 cm were employed. A sub-region of the DMD covering 128 × 128 micromirrors was used for light modulation. Prior to image acquisition, a fibre characterisation step was performed by a fibre characterisation unit that comprised a CMOS camera (C11440-22CU01, Hamamatsu Photonics, Shizuoka, Japan) for capturing the output speckle patterns after magnification by an objective (20×, 0.4 NA, RMS20X, Thorlabs, New Jersey, USA) and a tube lens (AC254-0100-A-ML, Thorlabs, New Jersey, USA). The focal plane of the camera was set to approximately 100μm away from the distal end of the fibre.

### 2.2 Multimode fibre characterisation

A real-valued intensity transmission matrix (RVITM)-based method was used for fibre characterisation with a DMD. The method has been detailed in previous studies [43], [44], here briefly, a Hadamard matrix H ∈ (−1, +1) with dimensions of N × N was generated in MATLAB, then two binary matrices $H_{1}=\left(H+1\right)/2$ and $H_{2}=\left(-H+1\right)/2$ were constructed based on H and combined as a binary matrix $\left[H_{1},H_{2}\right]$ ∈ (0, 1). All the columns of the matrix $\left[H_{1},H_{2}\right]$ were then converted into square patterns, which were sequentially displayed on the DMD whilst the intensity distributions of the output speckles at the distal fibre tip were captured. In our previous studies [43], [44], it was found that intensity changes from all the input modes (DMD micromirrors) to the output modes (camera pixels) could be modelled as: 

(1) $$\left[\begin{array}{ccc} I_{1}^{1} & \cdots & I_{1}^{2N}\\ \vdots & \ddots & \vdots \\ I_{m}^{1} & \cdots & I_{m}^{2N} \end{array}\right]=RVITM•\left[H_{1},H_{2}\right],$$

where $I_{m}^{k}$ is the intensity at the mth output mode when the kth binary Hadamard pattern is displayed as input, N is the total number of input modes, and • represents matrix multiplication. Then, by taking advantage of the properties of the Hadamard matrix, we have ${\left[H,-H\right]}^{T}={\left[H,-H\right]}^{-1}$, and the value of RVITM can be calculated via: 

(2) $$RVITM=\left[\begin{array}{ccc} 2I_{1}^{1}-I_{1}^{1} & \cdots & 2I_{1}^{2N}-I_{1}^{1}\\ \vdots & \ddots & \vdots \\ 2I_{m}^{1}-I_{m}^{1} & \cdots & 2I_{m}^{2N}-I_{m}^{1} \end{array}\right]•{\left[H,-H\right]}^{T},$$

As a result, a positive $rvit_{mn}$ (the intensity transmission constant linking the nth input mode and the mth output mode) indicates an increase of intensity at the mth output mode when the nth micromirror is switched ‘ON’. $rvit_{mn}$ can be further expressed as [44]: 

(3) $$rvit_{mn}=A_{mn}A_{R}cos\left(\theta_{mn}-\phi_{R}\right)$$

where $A_{mn}$ and $\theta_{mn}$ are the amplitude and phase at the mth output mode when only the nth micromirror is switched ‘ON’, $A_{R}$ and $\phi_{R}$ are the amplitude and phase of the output light field when all micromirrors are switched ‘ON’, respectively [44]. Eq. (3) suggests that switching ‘ON’ micromirrors with positive $rvit_{mn}$ values leads to constructive interference because the phases of output fields are in the range of [−π/2,π/2]. Furthermore, we ranked all the micromirrors descendingly with their $rvit_{mn}$ values and switched ‘ON’ the top 30% micromirrors in the optimal patterns to achieve the highest peak-to-background ratio for focusing at desired spatial locations at the distal end of the MMF, as we demonstrated in Ref. [44].

### 2.3 Image acquisition

After fibre characterisation, the fibre characterisation unit at the distal fibre tip was replaced by a PA imaging unit (Fig. 1), which comprised a single-element piezoelectric US transducer (V358, central frequency: 50 MHz; diameter: 0.25 inches, Olympus, Japan) and a cover slip as a sample holder that were integrated into a custom imaging chamber. An acoustic lens (LC4210, f=−25 mm, Thorlabs, New Jersey, USA) was attached to the active surface of the US transducer for focusing at the optical focal plane of the MMF. Both the US transducer and the distal end of the MMF were affixed to two 3D translation stages to facilitate precise alignment between the focus of the US transducer, the focal plane of the MMF and the imaging targets. The diameter of the acoustic focal area at the focal plane was estimated to be ∼ 50μm. Imaging targets, including carbon fibre phantoms and mouse RBCs, were placed on the sample hold located at the optical focal plane. The imaging chamber was filled with deionised water for acoustic coupling.

By displaying optimal DMD patterns, the excitation light was focused through the MMF and raster-scanned over the imaging targets. For PA imaging, the generated US waves were received by the US transducer, which were then amplified (SPA.1411, Sprectrum Instrumentation, Grosshansdorf, Germany), digitised by a data acquisition card (M4i.4420, sampling rate: 250 MHz, Sprectrum Instrumentation, Grosshansdorf, Germany) and transferred to a personal computer (Intel i7, 3.2 GHz) for processing and display. For fluorescence imaging, the excited fluorescence light was collected by the MMF and captured by a photodetector at the proximal fibre end (400−1000 nm, 10 MHz, APD410A/M, Thorlabs, New Jersey, USA) after reflected by a beam splitter (BS013, Thorlabs, New Jersey, USA) and spectrally filtered by a longpass filter (FEL0550, Thorlabs, New Jersey, USA). The received fluorescence signals were then sent to a second channel of the data acquisition card and transferred to the personal computer for processing. Synchronisation of the data acquisition, DMD pattern display, and laser firing was controlled by a functional generator (33600 A, Keysight, Santa Rosa, California) and a custom MATLAB program.

### 2.4 Experiments

Several experiments were performed to evaluate the performance of the imaging system. First, the focusing performance with the RVITM-based method was evaluated for two commercial MMFs: a STIN fibre with a core diameter of 105μm, and a NA of 0.22, and a GRIN fibre with a core diameter of 100μm, and a NA of 0.29 NA. Three performance metrics were compared: the enhancement factor (EF), the size of the focus and the power ratio. The EF was defined as the ratio of the average light intensity in the focal area over the average light intensity in the background; the size of the focus was defined as the full-width-at-half-maximum (FWHM) value of the intensity profile across the centre of the focus; the power ratio was defined as the fraction of total output light energy distributed in the focal area. We also studied the focusing performance through a GRIN fibre during fibre bending as it was reported that GRIN fibres are more resistant to bending [45]. The fibre was characterised when it was straight, then it was bent with varying angles, while corresponding EFs of two foci at central and side positions were recorded.

Second, to compare the imaging performance of the STIN and GRIN fibres, phantoms comprising networks of carbon fibre with a nominal diameter of 7μm were imaged over a 100 μm × 100 μm field-of-view with a scanning step size of 0.5μm. As the acoustic focus at the focal plane is smaller than the imaging area, the US transducer was carefully moved towards the carbon fibres using a translation stage so that the sensitive area of the ultrasound transducer covered the entire imaging field-of-view. However, the sensitivity of the ultrasound detection was degraded and the received PA signals were averaged over 16 repeated measurements for higher signal-noise-ratio (SNR).

Third, to study the dependency of imaging speed and spatial resolution on the scanning step size, PA imaging was performed with a carbon fibre network phantom. The phantom was placed in the focal plane of the focused ultrasound transducer to maximise the ultrasound detection sensitivity. PA images over a field-of-view of 50 μm × 50 μm were acquired with varying step sizes of 0.5, 1, 1.5, 2 and 2.5μm, corresponding to $100^{2},50^{2},34^{2},25^{2}$ and $20^{2}$ pixels per image. To estimate the lateral resolution, an edge spread function (ESF) was first obtained by averaging across profiles at 10 adjacent positions across an edge of a carbon fibre for each PA maximum intensity projections (MIP) image in the X-Y plane, and then a line spread function (LSF) was achieved by calculating the derivative of the ESF. The lateral resolution was calculated as the FWHM value of the Gaussian fit of the LSF. Similarly, as the axial resolution is independent of the scanning step size, it was estimated by profiles across an edge of a carbon fibre in the PA MIP image in the X-Z plane with a scanning step size of 0.5μm. To demonstrate the video-rate implementation of PAI through a MMF, the DMD was set to run at the uninterrupted mode with a frame rate of 22.7 and 47 kHz, respectively.

Fourth, the capability of the probe for PAI of biological tissue was evaluated by imaging *ex vivo* mouse RBCs with the GRIN fibre. Mouse blood was obtained from culled mice: these procedures involving mice were ethically reviewed and carried out in accordance with the Animals (Scientific Procedures) Act 1986 (ASPA) UK Home Office regulations governing animal experimentation. To maximise the PA signal strength, the field-of-view was set as 50 μm × 50 μm defined by the size of the acoustic focus. The scanning step size was varied from 0.5 to 2.5μm and PA signals were averaged over repeated measurements to increase the SNRs. As the laser energy of each ns-pulse decreased by half when the pulse repetition rate was increased from 22 kHz to 47 kHz, and RBCs generated weaker US signals than carbon fibres, the DMD was operated at 22.7 KHz.

Finally, the capability of dual-modal PA and fluorescence imaging was demonstrated with phantoms comprising carbon fibres and fluorescent microspheres over an area of 100μm × 100μm with a step size of 0.5μm. The carbon fibre had a nominal diameter of around 7μm and was placed on a coverslip. A drop of a solution containing 4μm fluorescent microspheres (TetraSpeck) was then added onto the coverslip and allowed to dry at room temperature prior to imaging. Images for the two modalities were acquired simultaneously with the same MMF (GRIN, 100μm in diameter, 0.29 NA) and the same excitation laser.

**Fig. 2:**
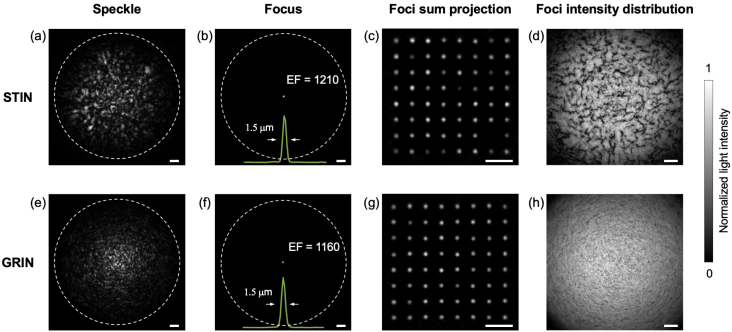
Focusing performance through step-index (STIN) and gradient-index (GRIN) multimode fibres. (a, e) Images of typical output speckles with random DMD patterns as inputs. (b, f) Images of optical foci when optimal patterns are used as inputs for STIN and GRIN fibres, respectively. The insets in (b) and (f) are lateral intensity profiles across the centres of the corresponding foci in (b) and (f). A series of foci images at different positions are shown in (c) and (g). (d) and (h) are the distributions of the peak foci intensities when an optical focus is scanned across the optical focal plane through the STIN and GRIN fibres, respectively. Scale bars: 10μm.

## 3 Results

### 3.1 Focusing performance

**Fig. 3:**
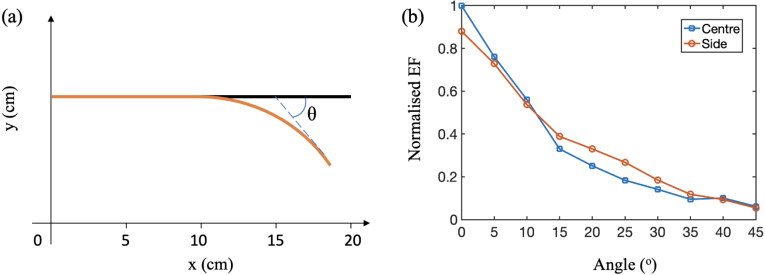
The evolution of focusing performance through a GRIN fibre with fibre geometry changes. (a) The schematic diagram of fibre geometry changes. Fibre characterisation was implemented when the fibre was straight (black) and the focusing performance was evaluated when bending the fibre from the middle point with an increasing angle. The final state with a bending angle of 45° was represented with the orange curve. (b) The evolution of focusing performance with increasing bending angle. EF, enhancement factor.

Fig. 2 shows the results of light focusing performance through MMFs. When a random DMD pattern was projected to the fibres, the outputs for both fibres are random-like speckles. However, the speckles with the STIN fibre was extended to a larger region compared to those with the GRIN fibre (Fig. 2a,e). Tight optical foci were generated with the RVITM method for both fibres, with an EF of 1210 for the STIN fibre and an EF of 1160 for the GRIN fibre (Fig. 2b, f). The focus sizes were the same for both fibres (1.5μm; Fig. 2b, f). The power ratios for the STIN and GRIN fibres were 10.6% and 9.1%, respectively. Fig. 2(c ,g) show the generated optical foci patterns at different spatial locations, and Fig. 2(d, h) show the peak intensity value distributions of the generated foci with a scanning step of 0.5μm for the two fibres. It is clearly seen that the peak intensities of the foci are much more uniformly distributed with the GRIN fibre compared to the STIN fibre, suggesting a higher image quality for the GRIN fibre. The focusing performance of the GRIN fibre to geometry changes is shown in Fig. 3. While the EF of foci at both central and side positions degraded gradually with the increase of the bending angle, the light foci were retained under a bending with an angle up to 45°.

**Fig. 4:**
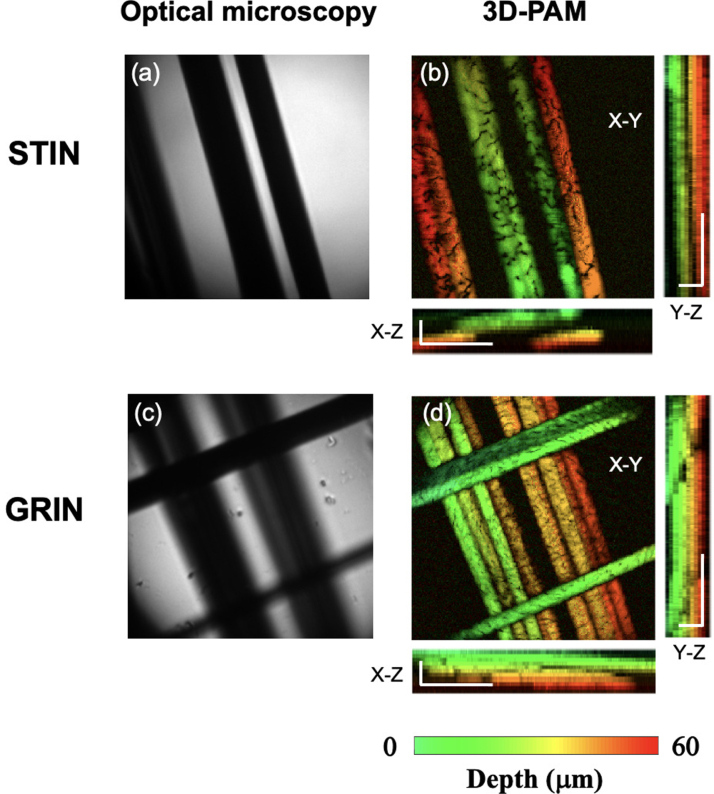
Photoacoustic (PA) imaging of carbon fibres with a step-index (STIN) and a gradient-index (GRIN) multimode fibres. (b) and (d) are PA maximum intensity projection images of a carbon fibre phantom in the X-Y, X-Z and Y-Z planes with the STIN and GRIN fibres, respectively, and (a) and (c) are corresponding optical microscopy images. Depth information in the PA images are coded with false colours. Scale bars in all the X, Y and Z directions represent 30μm.

### 3.2 Photoacoustic imaging of carbon fibre phantoms

Fig. 4(a) and (c) show conventional optical microscopy images of the carbon fibre phantoms with the same field-of-view acquired in transmission mode. It can be seen that conventional optical microscopy was able to provide 2D visualisation and thus it was not able to spatially resolve individual carbon fibres in some overlapped regions. Fig. 4(b) and (d) show the MIPs of PA images in the X-Y, Y-Z and X-Z planes for both the two phantoms. PA imaging was able to provide 3D visualisation of individual carbon fibres with high fidelity; the depth information was provided by the time-resolved PA signals from each scanning location and was coded with false colours. Compared to the STIN fibre (Fig. 4b), PA images achieved with the GRIN fibre (Fig. 4d) showed a more uniform pixel amplitude distribution across the carbon fibres regions, which was attributed to the more uniform foci intensity distribution as shown in Fig. 2(h).

**Fig. 5:**
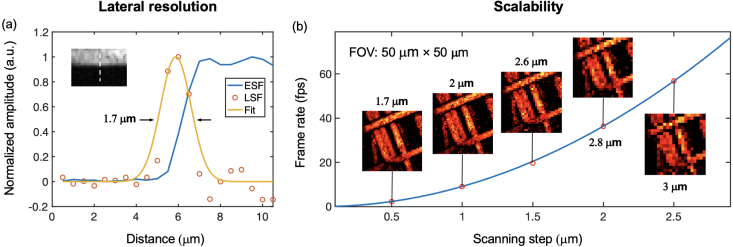
Scalability of the multimode fibre-based imaging system demonstrated with imaging of a carbon fibre phantom. Method for lateral resolution measurement is illustrated in (a), and the scalability on the spatial resolution and imaging speed is shown in (b). With an imaging field-of-view of 50 μm× 50 μm, and the digital micromirror device running at 22.7 kHz, the frame rate of image acquisition increases with the scanning step size from 2.27 to 56.75 frames per second (fps), leading to the degradation of the measured lateral resolution from 1.7 to 3μm. ESF, edge spread function; LSF, line spread function; FOV, field-of-view.

Since scanning of the light focus was realised via optical wavefront shaping, the scanning step size and field-of-view could be easily controlled by displaying corresponding DMD patterns, which enabled high scalability on the achievable imaging frame rate and spatial resolution as shown in Fig. 5. The GRIN fibre was used for PA imaging of a carbon fibre phantom for the measurement of the spatial resolution. With the DMD running at 22.7 kHz, the rate of the PA image acquisition varied from 2.27 to 56.75 fps with corresponding scanning step size increased from 0.5 to 2.5μm. The lateral resolution with a scanning step size of 0.5μm was estimated as 1.7μm (Fig. 5a), which is consistent with the size of the optical focus through the same MMF (1.5μm). With the increase of the scanning step size, the lateral resolution worsened gradually from 1.7 to 3μm (Fig. 5b). The estimated axial resolution was ∼ 27μm, which was consistent with the frequency bandwidth of the US transducer (27–63 MHz) [46]. Increasing the scanning step size to 1.5μm virtually retained the main features of the carbon fibres and allowed a frame rate of ∼ 20 fps with the DMD running at 22.7 kHz. Videos 1 and 2 are recordings with a smart phone camera showing the real-time acquisition and display of PA images of a carbon fibre phantom that was translated manually using a translation stage during image acquisition. Videos 1 and 2 were acquired when the DMD was operated at 22.7 kHz and 47 kHz, respectively. The display speed was limited by the Matlab program for data acquisition, processing and display that had a time interval of ∼ 0.1 s for displaying sequential frames, and the function of saving raw US signals during image display. A bandpass filter (15–55 MHz cutoff frequency) was employed for offline processing to improve image quality as detailed in supplementary material (Fig. S1). Four individual image frames after offline processing from the raw US signals obtained during the acquisition of Video 2 are shown in Fig. S2. The image acquisition time per frame, calculated by the ratio of the pixel count (1156) to the scanning rate (47 k), was 0.0246 s, leading to an image acquisition rate of ∼40 fps.

**Fig. 6:**
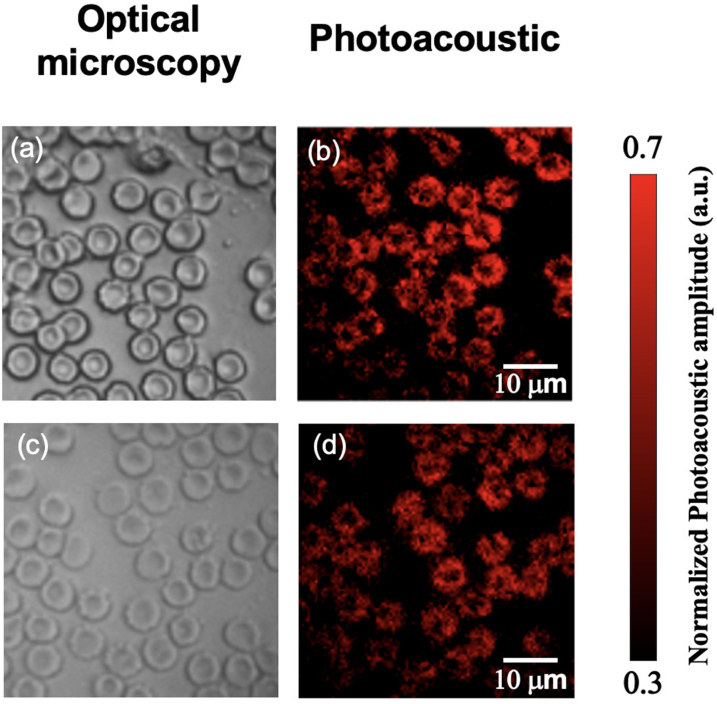
Photoacoustic imaging of mouse red blood cells *ex vivo*. (a) and (c) are optical microscopy images of mouse red blood cells, (b) and (d) are corresponding photoacoustic images of the same regions. Photoacoustic signals were averaged over 16 consecutive measurements and the scanning step size was 0.5μm. a.u., arbitrary unit. Scale bars: 10μm.

### 3.3 Photoacoustic imaging of mouse red blood cells

The capability of imaging biological tissues was demonstrated with mouse red blood cells. Fig. 6(b) and (d) show PA MIP images in the X-Y plane achieved with averaging across 16 consecutive frames and a scanning step size of 0.5μm. The bi-concave structures of RBCs were clearly visualised and corresponded well to the optical microscopy images of the samples over the same field-of-view (Fig. 6a, c). The image quality is comparable to those of the RBCs images obtained with benchtop PA microscopes reported in literature [47], [48]. Each image covered an area of 50μm × 50μm with 100 by 100 pixels, leading to an acquisition time of 7 s for each image with the DMD running at 22.7 kHz. The acquisition time can be shortened by increasing the scanning step size and reducing the number of signal averages, however, at the expense of the image fidelity (Fig. S3). Video 3 is a recording with a smart phone camera showing the real-time acquisition and display of PA images of a RBC translated manually using a translation stage. The scanning step size was 1.5μm and no signal averaging was used to allow a frame rate of ∼20 fps. Four individual image frames after offline processing from the raw US signals obtained during the acquisition of Video 3 are shown in Fig. S4. Further, with the use of time-resolved PA signals, RBCs at different depths were also distinguished in the 3D-PA image with depth information colour-coded (Fig. S5).

**Fig. 7:**
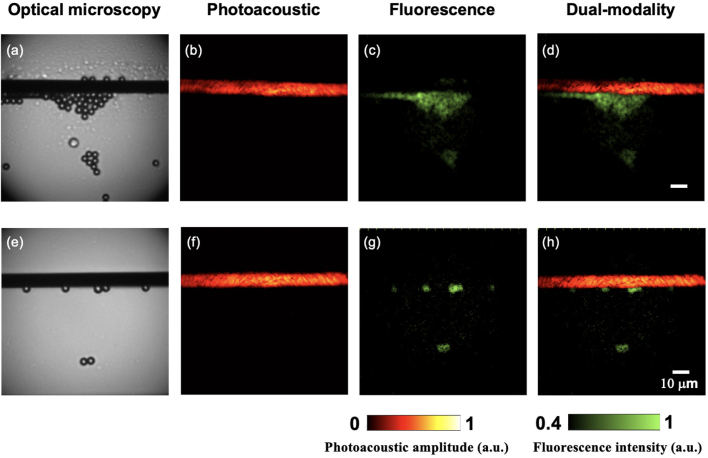
Dual-modal photoacoutic and fluorescence imaging of phantoms comprising carbon fibres and fluorescent microspheres. PA signals for phantom on the top row were averaged across 4 consecutive measurements, and on the bottom, across 16 consecutive measurements. Scale bar: 10μm.

### 3.4 Simultaneous photoacoustic and fluorescence imaging

As shown in Fig. 7, PA MIP images in the X-Y plane clearly visualised the carbon fibres with high fidelity and corresponded well to the corresponding optical microscopy images. On the other hand, fluorescence imaging highlighted the fluorescent microspheres as expected. However, due to the limited spatial resolution, individual fluorescent microshperes could not be resolved clearly at the overlapping regions (Fig. 7c).

## 4 Discussion

In this study, we developed a high-speed dual-modal forward-viewing imaging probe that integrated simultaneous PA and fluorescence imaging capabilities at the tip of an ultrathin MMF. The performance of the imaging system in terms of spatial resolution and imaging speed was evaluated with carbon fibre phantoms, and the capability of imaging biological tissues with PAI was demonstrated by visualising mouse RBCs with a high fidelity comparable to those achieved with benchtop PA microscopes [47], [48]. Wavefront shaping with a DMD allowed raster-scanning of a focused laser beam with varying step sizes, so that the imaging speed is scalable with the scanning step size, ranging from 2–57 fps over an area of 50μm × 50μm. The ability of high-speed imaging is attributed to the use of a DMD for light modulations. Here the frame rate of the DMD was set to be 22.7 kHz, which corresponds to scanning 22.7 k image pixels per second. To our best knowledge, the fastest pixel-scanning rate for MMF-based PAE in literature was 60 Hz and it took ∼30 s to acquire a 1800-pixel image (∼0.03 fps) [42]. In comparison, our system enabled an imaging frame rate of ∼20 fps over a similar field-of-view and a similar scanning step size. The highest frame rate that we achieved was ∼40 fps with a carbon fibre phantom when the DMD was operated at an uninterrupted mode of 47 kHz.

The speed of high-quality PA imaging of RBCs is mainly limited by the decrease of laser pulse energy when the pulse repetition rate of the laser is larger than 10 kHz. Although the RVITM method had a higher usage ratio of laser energy compared to holographic approaches [49], the laser pulse energy transmitted through the optical fibre was ∼100 nJ at a pulse repetition rate of 10 kHz and declined to 62 nJ at 22.7 kHz and 30 nJ at 47 kHz. Further, the focusing enhancement obtained in this work was lower than that reported with holographic approaches with a larger number of input modes [29] and thus resulted in a lower power in light foci. Thus, signal averaging was employed for improving the SNRs of the PA signals from RBCs, which slowed down the imaging speed for high-fidelity biological tissue imaging. Apart from improving the laser energy, the employment of a larger number of independent micromirrors can improve the focusing enhancement [50], leading to a higher SNR, however, at the expense of a longer fibre characterisation time.

Compared to multi-core fibre bundle-based PA endoscopy probes [22], [24], the use of MMFs reduced the probe size to 140μm in diameter and improved the lateral resolution to 1.7μm. However, the use of MMFs also involves the challenges of fibre bending-induced system instability as changes in the fibre geometry can lead to substantial changes in the light transmission characteristics, leading to degradation of the focusing performance as demonstrated in Fig. 3. Although a number of approaches have been reported to address this challenge, such as TM reconstruction based on the known fibre geometry [51] and GPU accelerated real-time characterisation [52], achieving a fully flexible MMF-based imaging probe with wavefront shaping remains challenging. In this study the image quality with a GRIN fibre is found to be higher compared to that with a STIN fibre due to the more uniform distribution of the peak energies of the optical foci that is associated with the GRIN fibre. In a recent study, it was reported that GRIN fibres have a higher resistance to geometry changes on the imaging performance compared to STIN ones [45]. In future studies, the PA and fluorescence imaging performance will be studied with a GRIN fibre probe integrated into a medical needle and as such the geometry of the fibre could be relatively unchanged. Apart from MMF, several novel optical fibres have been investigated for higher resistance to geometry changes of the fibre. A conformationally invariant multi-core fibre with twisted cores was reported for a flexible endoscope, which allowed the light focusing with geometry changes of the fibre for digital confocal two-photon imaging [53]. A disordered glass-air Anderson localisation optical fibre was also demonstrated to be resistant to fibre bending for imaging transmission applications using deep learning [54].

In this study, a conventional piezoelectric US transducer was used for US detection in a transmission mode, which restricted its use for endoscopic applications. In the future, a miniature US sensor could be attached to the MMF to receive PA signals in a reflection mode. This sensor could be a miniature piezoelectrical US sensor [55], a fibre-optic Fabry–Perot sensor [56] or a micro-ring resonator US sensor [57].

## 5 Conclusions

In summary, we developed a dual-modal PA and fluorescence microscopy probe based on a MMF. With a fast DMD used for wavefront shaping, high-fidelity PA images of carbon fibres and mouse RBCs were acquired at high-speed. Dual-modality imaging capability was demonstrated with phantoms consisting of carbon fibres and fluorescence microspheres. With further miniaturisation of the US detector for imaging in a reflection mode, the imaging platform has the potential to guide various minimally invasive procedures such as tumour biopsy, and nerve blocks, by providing micro-structural, functional and molecular information of tissue at high spatial resolution in real-time from within a medical needle.

## Declaration of Competing Interest

The authors declare that they have no known competing financial interests or personal relationships that could have appeared to influence the work reported in this paper.T. V is co-founder and shareholder of Hypervision Surgical Ltd, London, UK. He is also a shareholder of Mauna Kea Technologies, Paris, France.

## Appendix A Supplementary data

Video S1 Photoacoustic imaging of a carbon fibre phantom at 20 frames per second.

Video S2 Photoacoustic imaging of a carbon fibre phantom at 40 frames per second.

Video S3 Photoacoustic imaging of a mouse red blood cell at 20 frames per second.

MMC S1 Signal processing pipeline and additional examples of photoacoustic images obtained with varying imaging parameters.
