# Supplementary material for: Video-rate dual-modal photoacoustic and fluorescence imaging through a multimode fibre towards forward-viewing endomicroscopy
Source: Photoacoustics. 2021 Dec 31;25:100323. doi: 10.1016/j.pacs.2021.100323 (PMC8741494; doi:10.1016/j.pacs.2021.100323)
Supplement: MMC S1 — Signal processing pipeline and additional examples of photoacoustic images obtained with varying imaging parameters. [file mmc4.pdf]

 Click here to view linked References

Supplementary materials for "Video-rate dual-modal photoacoustic and fluorescence imaging through a multimode fibre towards forward-viewing endomicroscopy"

Tianrui Zhao[a], Michelle T. Ma[a], Sebastien Ourselin[a], Tom Vercauteren[a], Wenfeng Xia[a,*]
[a]School of Biomedical Engineering and Imaging Sciences, King's College London, 4th Floor, Lambeth Wing St Thomas' Hospital, London SE1 7EH, United Kingdom

*Wenfeng Xia, wenfeng.xia@kcl.ac.uk

1 Photoacoustic signal processing

The signal processing pipeline is shown in Fig S1. Raw ultrasound signals were first frequency filtered (15-55 MHz bandpass filter) to improve the signal-to-noise ratios, and the maximum of the absolute values of the signal after Hibert transformation was then used to construct a single pixel of an maximum intensity projection image. Signal averaging across varying number of repeated measurements was performed to further improve the SNR depending on the imaging target.

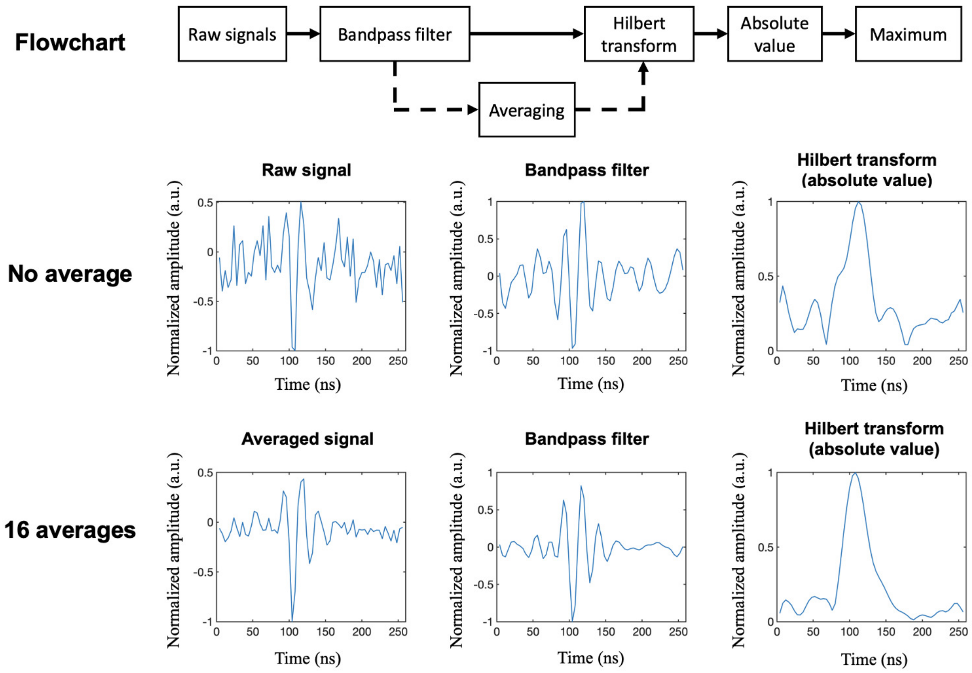

Fig S 1 Photoacoustic signal processing pipeline including bandwidth filter, Hilbert transform, and signal averaging.

2 Video-rate photoacoustic imaging

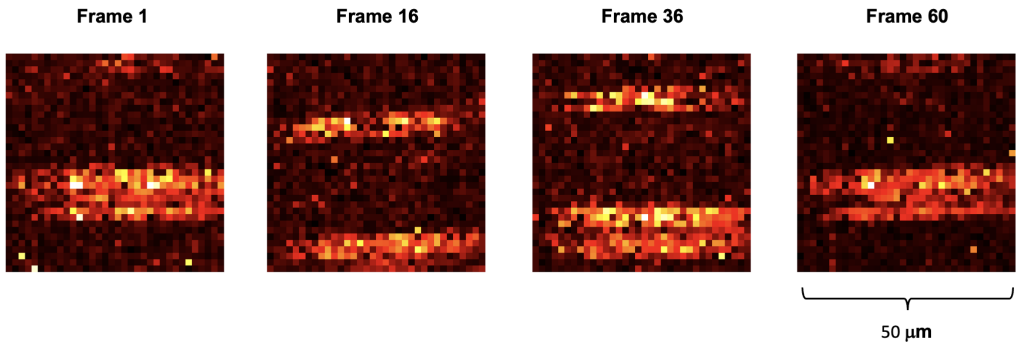

Fig S 2 Photoacoustic image frames after offline processing from the raw ultrasound signals obtained during the acquisition of Video 2 when the carbon fibres were translated manually using a translation stage.

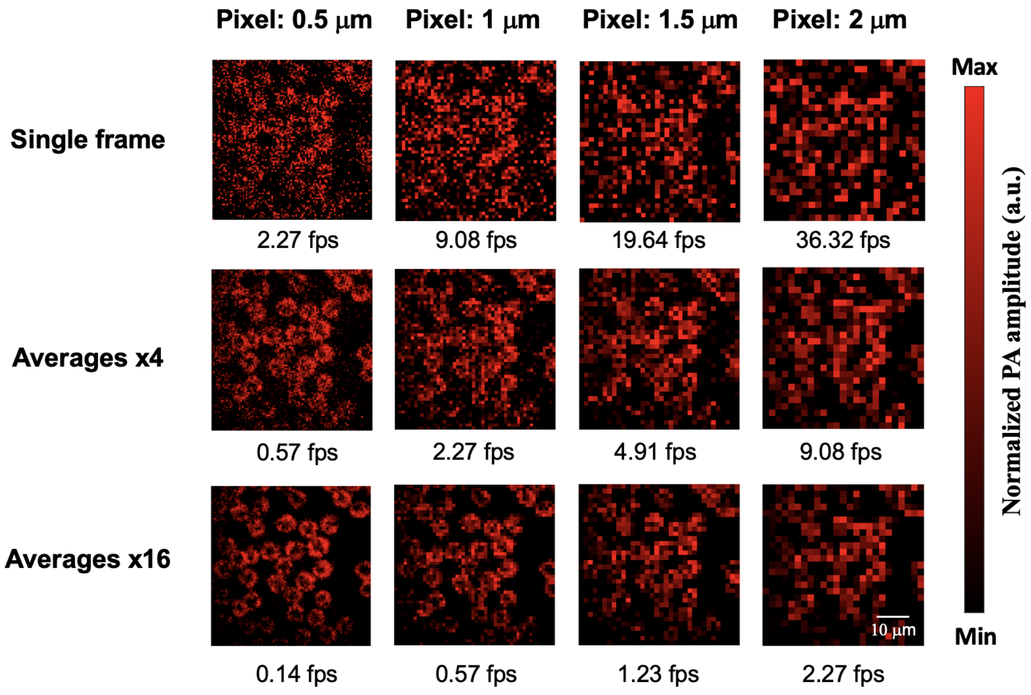

Fig S 3 Scalability of the multimode fibre-based imaging system demonstrated with imaging of mouse red blood cells.

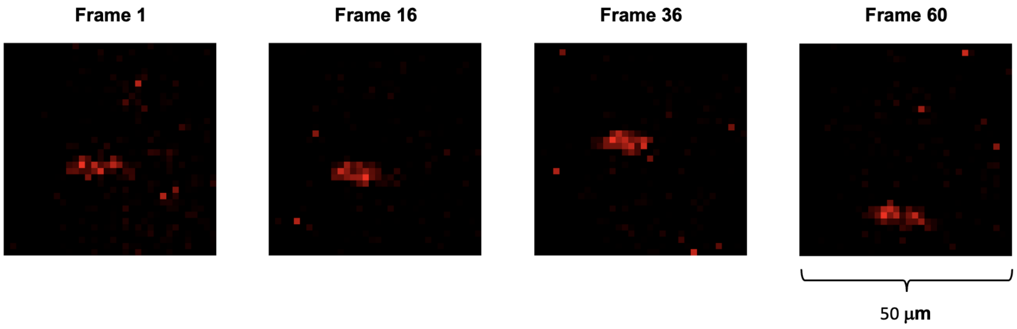

Fig S 4 Photoacoustic image frames after offline processing from the raw ultrasound signals obtained during the acquisition of Video 3 when the mouse red blood cell was translated manually using a translation stage.

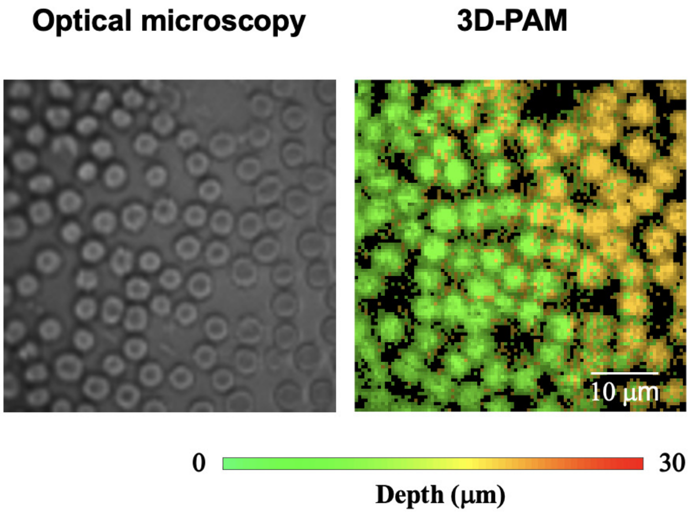

Fig S 5 3D-PA imaging of red blood cells with depth information in the PA maximum intensity projection images coded with false colours.
